# Co-creation of the Global Patient Experience Data Navigator: a multi-stakeholder initiative to ensure the patient voice is represented in health decision-making

**DOI:** 10.1186/s40900-023-00503-9

**Published:** 2023-10-12

**Authors:** Tom Willgoss, Omar A. Escontrias, Carole Scrafton, Elisabeth Oehrlein, Victoria Livingstone, Fiona C. Chaplin, Maddalena Benivento, Hayley Chapman, Nicholas Brooke

**Affiliations:** 1 Patient Centered Outcomes Research, Roche, Hexagon Place, Falcon Way, Welwyn Garden City, UK; 2 Escontrías, National Health Council, 1730 M St. NW, Suite 650, Washington, DC 20036 USA; 3 Flutters and Strutters Patient Advocacy Organisation, Sunderland, UK; 4 Applied Patient Experience, LLC, 2201 Wisconsin Ave NW, Suite 200, Washington, DC 20007 USA; 5 Global Patient Engagement, Medical Affairs, Gilead Sciences Europe Ltd, Roundwood Avenue, Stockley Park, Uxbridge, UK; 6 Twist Medical Ltd, 125 Howard Avenue, Suite 530, Burlingame, CA 94010 USA; 7 Patient Focused Medicines Development (PFMD), The Synergist, Avenue Louise 231, 1000 Brussels, Belgium

**Keywords:** Patient engagement, Patient experience data, Public and patient involvement, Patient-generated data, Medical, pharmaceutical, and technology development, Healthcare, Patient-reported outcomes, Patient-preference studies, Patient-centered care

## Abstract

**Background:**

Putting patients’ needs and priorities at the forefront of healthcare initiatives and medical product development is critical to achieve outcomes that matter most to patients. This relies on the integration of early, meaningful patient engagement (PE) to learn what is important to patients, and collection of representative patient experience data (PXD). The increased number of PE/PXD efforts across global regulatory, health technology assessment, and healthcare systems is an important step forward to deliver improved health outcomes for patients. However, these initiatives are fragmented and lack integration, which is necessary to maximize efforts and reduce burden on patients. To overcome these challenges, the Global Patient Experience Data Navigator has been co-created by Patient Focused Medicines Development to provide practical resources that can facilitate and optimize PXD generation, collection, analysis, and dissemination for patient benefit and aims to be applicable across all therapeutic areas for all stakeholders.

**Methods:**

Co-creation of the Navigator took place through an iterative process of validation and formalization driven by a diverse, multi-stakeholder working group with individuals who have varying knowledge/experience in PE/PXD.

**Results:**

A series of workshops took place to conduct a gap analysis, develop a taxonomy model, and integrate existing frameworks. The collective insights led to the development of the Navigator consisting of four specific tools in the form of downloadable templates, which can be used to: (1) prioritize outcomes that matter most to patients and their caregivers; (2) select appropriate measurement methods for these outcomes; (3) identify when and why PXD is used throughout the product development cycle for each stakeholder; (4) identify when and why PXD is used throughout the healthcare process for each stakeholder. A public consultation was carried out to collect user feedback before the Navigator was made publicly available in December 2022.

**Conclusion:**

To our knowledge, the Global Patient Experience Data Navigator is the only publicly available toolkit developed with a multi-stakeholder and disease-agnostic approach providing taxonomically grouped resources to optimize the collection and collation of PXD for patient benefit. Future work will aim to further engage patients by adding a PE dimension to the Navigator.

**Supplementary Information:**

The online version contains supplementary material available at 10.1186/s40900-023-00503-9.

## Abstract (plain language summary)

Engaging patients at the start of healthcare and medical product development projects can help better understand their experiences and what is most important to them. Ultimately this will achieve the best outcomes. However, if not carefully planned, projects that engage patients can lead to inefficiencies, such as patients being asked for the same information repeatedly. The collection of patient experience data—information related to patients’ experiences, needs, and priorities—also needs to be carefully managed. To help solve this problem, Patient Focused Medicines Development developed a publicly available “toolkit” called the Global Patient Experience Data Navigator. The Navigator has downloadable templates that can be filled in and used for projects in any disease area and by anyone collecting patient experience data. To represent different perspectives, individuals with a range of experiences and understanding of patient engagement projects worked together to co-create the toolkit. Several meetings took place to understand what the toolkit needed to do and to help provide a structure for the templates. There are four templates in the toolkit. These can be used to: (1) prioritize outcomes that matter most to patients and their caregivers; (2) select the best ways of measuring these outcomes; (3) identify when, why, and by whom patient experience data is used throughout the product development cycle; and (4) identify when, why and by whom patient experience data is used throughout the healthcare process. Future work will utilize public feedback to make the toolkit more user-friendly and provide education on how the Navigator can be used.

**Supplementary Information:**

The online version contains supplementary material available at 10.1186/s40900-023-00503-9.

## Introduction

The value of engaging patients in medical product development (including pharmaceutical products and medical devices/technologies) and healthcare decision-making processes from the outset, to fully understand patient experiences and unmet needs, is increasingly recognized by healthcare stakeholders globally [1–5]. The idea that patients are collaborators calls for their participation at every step (of product development and healthcare decision-making processes), to optimize incorporation of patient insights, along with involving all other stakeholders [6]. For example, in clinical trials, earlier patient participation can influence decision-making from the outset and steer the choice of outcomes by measuring those that matter most to patients including those associated with wellbeing and daily life, such as patient-centered core impact sets (PC-CIS), as opposed to including only conventional clinical measures [7–9].

Consequently, there has been a call to action for multi-stakeholder collaboration to drive productive and well-planned patient engagement (PE) initiatives, to gain insights from patients, caregivers, and others affected by a disease or condition [2]. PE initiatives include any activities that enable the active and meaningful involvement of patients in developing medicines and healthcare management, for example active collaboration in the governance, priority setting, and conduct of research, as well as in summarizing, distributing, sharing, and applying its resulting knowledge. Such initiatives may lead to the collection of patient experience data (also referred to as PED and both terms are used interchangeably, but PXD is used in this article for internal consistency with other PFMD led projects), defined by the US Food and Drug Administration (FDA) as any information that captures patients’ experiences, perspectives, needs, and priorities related to (but not limited to) their disease symptoms, treatment/treatment preferences, outcome, and impact on functioning/daily life [10, 11].

Historically, the approaches and methodologies for the collection and organization of PXD are fragmented, lack integration and overall observation or analysis, and have led to duplication of efforts, often putting an unnecessary burden on patients or leading to inefficiencies in the healthcare system [1, 2, 4]. In addition, the increased number of PE and PXD activities generates new activities and practices by all parties involved [12]. Still, these are not always collated or managed efficiently to maximize patient benefit. To help address this, Patient Focused Medicines Development (PFMD) has developed the Global Patient Experience Data Navigator, part of PFMD’s Patient Engagement Management Suite, which serves as a practical resource to provide easily accessible tools to help plan and execute a wide range of PXD projects, and with a commitment to putting the patient voice at the centre of such initiatives [13, 14].

The Navigator provides clarity and structure to PXD collection, collation, and dissemination, with the needs of multiple stakeholders in mind, including patient advocates, patients, caregivers, the life science industry, health technology assessment (HTA) organizations, payors, healthcare professionals, regulators, researchers, funders, and those in clinical practice. Importantly, the Navigator was designed using a disease-agnostic approach and, therefore, aims to be globally applicable across a wide range of disease areas and PXD projects. Such projects might include PXD data collection by regulatory/HTA agencies to help support guidance for the inclusion of PXD in submissions, as well as projects initiated by healthcare providers directly in clinical practice for quality improvement; by patient organizations gathering data around health disparities; by payors to drive priority setting and reimbursement decision-making; and by life science/medical technology companies to drive value propositions [2]. Although these projects may differ in their overall goals, the processes, questions, and methodologies used to collect PXD are often similar. In this manuscript, we present data relating to the development and design of the Navigator in terms of the methodology used, along with preliminary results of a public consultation targeting potential users of the Navigator and their assessment of the pilot version. Development of the Navigator was part of a wider project by PFMD working toward accelerating and optimizing patient health outcomes through the union of PE and PXD across research and healthcare systems.

## Methods

### The development process

**Fig. 1:**
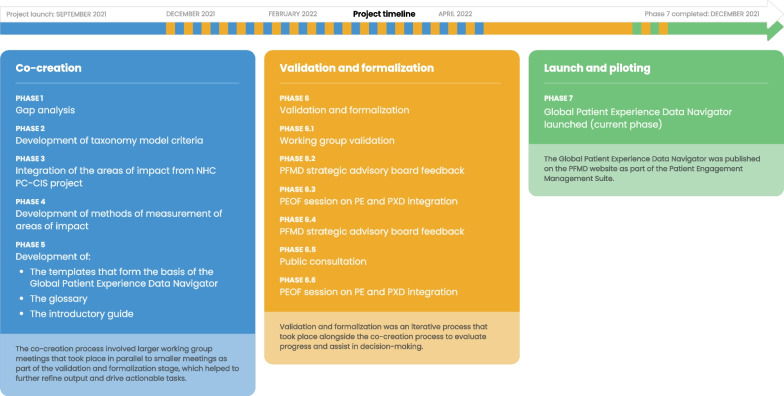
Process for the development of the Global Patient Experience Data Navigator. A schematic showing the co-creation and validation/formalization stages of the development process and the seven phases that comprised this, with a timeline to illustrate that the validation/formalization phases took place alongside the co-creation meetings. *NHC* National Health Council; *PC-CIS* patient-centered core impact sets; *PE* patient engagement; *PFMD* Patient Focused Medicines Development; *PXD* patient experience data

The Global Patient Experience Data Navigator was developed using a framework of co-creation, validation, and formalization, subdivided into seven distinct phases that included over 306 people representing more than 50 organizations. The co-creation process involved a series of working group (WG) meetings that took place in parallel to smaller meetings, workshops, and discussions, as part of the validation and formalization phase (Fig. 1). There was a strong commitment to including patient and public involvement (PPI) from the outset—with 10 and 6 patient representatives as members of the WG and steering committee (SC), respectively. The GRIPP2 short form reporting checklist was adhered to and is provided as Additional file 1.

### Co-creation, validation, and formalization

Co-creation of the Navigator included input from numerous WG and steering committee (SC) meetings, webinars, surveys, and open consultations. A multi-stakeholder WG of 24 individuals from diverse backgrounds representing patients, patient organizations, regulators, healthcare professionals, pharmaceutical companies, and external experts, was established with guidance from an SC. The WG had varying levels of knowledge and expertise in PE and PXD or could provide first-hand experience of PXD as a patient or patient representative. The SC included 11 individuals with expertise in PXD and/or patient engagement. SC members contributed to the Navigator in an advisory capacity and were updated regularly on its progress. A full list of the WG and SC members and their affiliations is provided in Additional file 2: Table S1. 

A series of formal WG meetings occurred during the initial co-creation phase. The focus of the WG meetings was to conduct a gap analysis and better understand the unmet needs in PXD, to develop a taxonomy model of PXD, and to integrate existing frameworks. Alongside these larger meetings, a series of ad-hoc smaller meetings and discussions took place to help further refine decision-making. In parallel with the WG meetings, a process of validation and formalization took place. Specifically, validation refers to achieving consensus among stakeholders regarding the content and format of the tool. This consensus was established through discussions in both group settings and during individual interactions and ensured that all participants could voice their opinions, and express agreement or disagreement. This comprised meetings, events, and activities with defined objectives and outputs to help refine the decision-making at each stage of the Navigator’s co-creation. To help understand stakeholder views on PE and PXD and further inform the project, a survey was conducted at the Patient Engagement Open Forum in April 2022, and the opinions/results were collated. Key resources used to develop the Global Patient Experience Data Navigator are outlined in Additional file 3: Table S2.

### Public consultation on the draft Global Patient Experience Data Navigator

A public online survey-based consultation was carried out from June 10 to September 17, 2022, to provide a better understanding of potential user demographics, expertise, and interaction (language, structure, how readily it might be integrated into existing processes), as well as potential uses and additional tools that might be of value to support the Navigator’s use and implementation. A set of questions for the survey was established based on experience developed from a previous PFMD project and public consultation [15]. The survey merged quantitative and qualitative methods, giving respondents the chance to rate the tool and support their choices with more extensive and qualitative answers. The target audience was broad and included anyone interested in PE and PXD. A progressive approach was adopted for dissemination, first reaching out to the WG and SC members, and then targeting a wider audience through a mailing list and social media campaign. All data was analysed separately by two independent researchers and plotted using relevant charts/graphs to provide maximum data transparency (all N values provided). At the time of publication, a link to the public consultation remains active on the current Global Patient Experience Data Navigator download page, as data will continue to be collected and used to refine the tool going forward.

### Co-creation results

Although the WG meetings provided the main structure for the project, if all of the channels that were used to develop the Navigator are accounted for, including WG and SC meetings, PFMD strategic advisory board meetings, Patient Engagement Open Forum, and public consultations, the total number of individuals who contributed to the overall development of the Global Patient Experience Data Navigator was 306. From these contributors, a total of 512 insights were collated and examined as part of the development process. Hence, the opinions of patient representatives and patients were included in planning and decision-making from the outset.

### Summary of the WG meetings

In total, seven WG meetings took place between September 2021 and June 2022 as part of phases 1–5 of the Navigator’s development (Fig. 1). In addition to the WG meetings, 11 smaller meetings were conducted between October 2021 and November 2021 to help refine and finalize outputs from the larger meetings. A summary of the WG meeting objectives and key outputs is provided in Additional file 4: Table 1.

The first WG meeting took place in September 2021 and focused on establishing the need for a global PXD taxonomy, as well as the criteria for a possible taxonomy model. It was agreed that the taxonomy should be comprehensive, extensible, and explanatory, capturing the ideal, rather than the current, state of PXD, and that diversity, cultural factors, and accessibility would also be considered, with a focus beyond research and development processes. WG consensus was that a suitable taxonomy model would contain four main sections addressing (1) WHAT: highlight the experiences that are most important to patients; (2) HOW: review the approaches and methodologies available (and identify gaps) to measure these patient experiences; (3) WHO: identify the stakeholders that are using PXD; (4) WHEN and WHY: consider when and why stakeholders are using these data. These discussions aimed to define the most important and meaningful needs to patients, building on what was already set out in an early version of the National Health Council Impact Taxonomy [9].

Developing the sections of the toolkit was the main objective of the WG meetings, taking each section at a time to establish all the necessary fields and using an iterative approach to refine the output with further external discussions. A glossary and an introductory guide were also developed.

### Validation and formalization

Alongside the WG meetings (i.e., phases 1–5 [Fig. 1]), and as part of phase 6 of the development process, an iterative process of validation and formalization took place. The aim of these meetings was to finalize decision-making and keep track of each section's progress. For ease of reporting and to distinguish between the different meetings and events, this phase has been subdivided (into phases 6.1–6.6). A summary of the meetings and key objectives/outputs for phase 6 is provided in Additional file 5: Table 2.

**Fig. 2:**
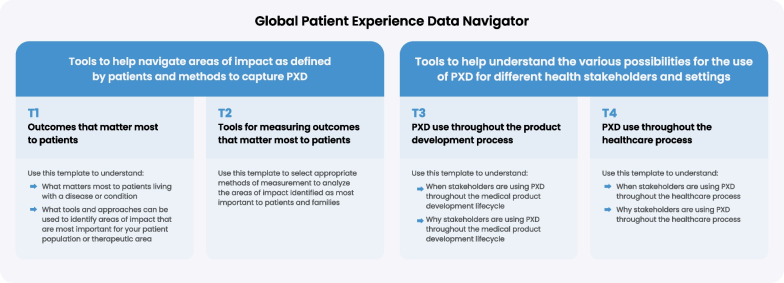
Schematic to describe the four tools that make up the Global Patient Experience Data Navigator. The four tools cover the whole lifecycle of PXD and each tool takes the format of a template that can be downloaded and filled in. *PXD* patient experience data

Phase 6.1 focused on creating the main resources for the Global Patient Experience Data Navigator, i.e., the practical tools to facilitate PXD collection. The Navigator comprises six sections: an Introduction, four tools for four different purposes covering the whole lifecycle of PXD, which take the format of a table or template and can be downloaded and completed (Fig. 2), and a glossary. The four tools are: (T1) outcomes that matter most to patients; organized to capture areas of impact, specific impact, prioritization approaches, and patient-focused listening activities; (T2) tools for measuring the outcomes that matter most to patients, organized to capture tools and appropriate methods for measuring these outcomes; (T3) PXD use throughout the product development process, organized to capture the various stakeholders and examples of how PXD is used in the medical product development process; (T4) PXD use throughout the healthcare process, organized to capture relevant stakeholders and examples of how PXD is used in the healthcare development process. The Global Patient Experience Data Navigator can be downloaded from the website [19]. Phase 6.2 focused on disseminating the Navigator to widen its reach (detailed in Additional file 5: Table 2).

### Stakeholder views on PE and PXD: value, challenges, and needs

**Fig. 3:**
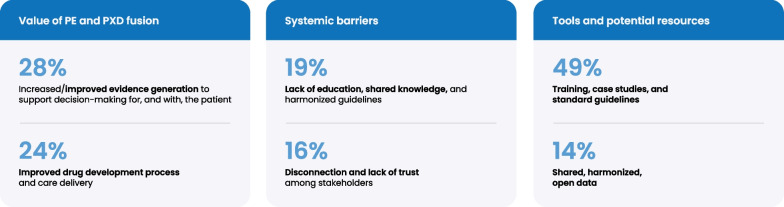
Results of a survey completed by 73 respondents from a range of stakeholder groups. The survey was completed during the Patient Engagement Open Forum in April 2022. Attendees were invited to answer questions about the value of merging PE and PXD initiatives. *PE* patient engagement; *PXD* patient experience data

Phase 6.3 took place during the Patient Engagement Open Forum on April 12, 2022, and addressed the concept of PE and PXD fusion, which refers to the need for meaningful PE when designing initiatives to generate, collect, and use PXD. A survey was completed by 73 respondents, from a range of stakeholder groups who were asked three questions: (1) Where do you see the value in PE combined with PXD for each stakeholder group? (2) What are the barriers to integrating PE into PXD evidence design and generation? (3) What support, tools, and resources are needed to strengthen the use and implementation of PE and PXD fusion? The results are summarized in Fig. 3.

When asked about the value of PE and PXD for each stakeholder group, 28% highlighted the need for increased/improved evidence generation to support decision-making for and with the patient, and 24% highlighted improved drug development process and care delivery. In terms of systemic challenges in achieving effective PE and PXD, 19% cited a lack of education, shared knowledge, and the need for harmonized guidelines, and 16% cited disconnection and lack of trust among stakeholders. With regard to the tools and potential resources for PE and PXD, 49% noted the need for training, case studies, and standardized guidelines, and 14% commented on the need for shared, harmonized, open data.

### Public consultation on the draft Global Patient Experience Data Navigator

**Fig. 4:**
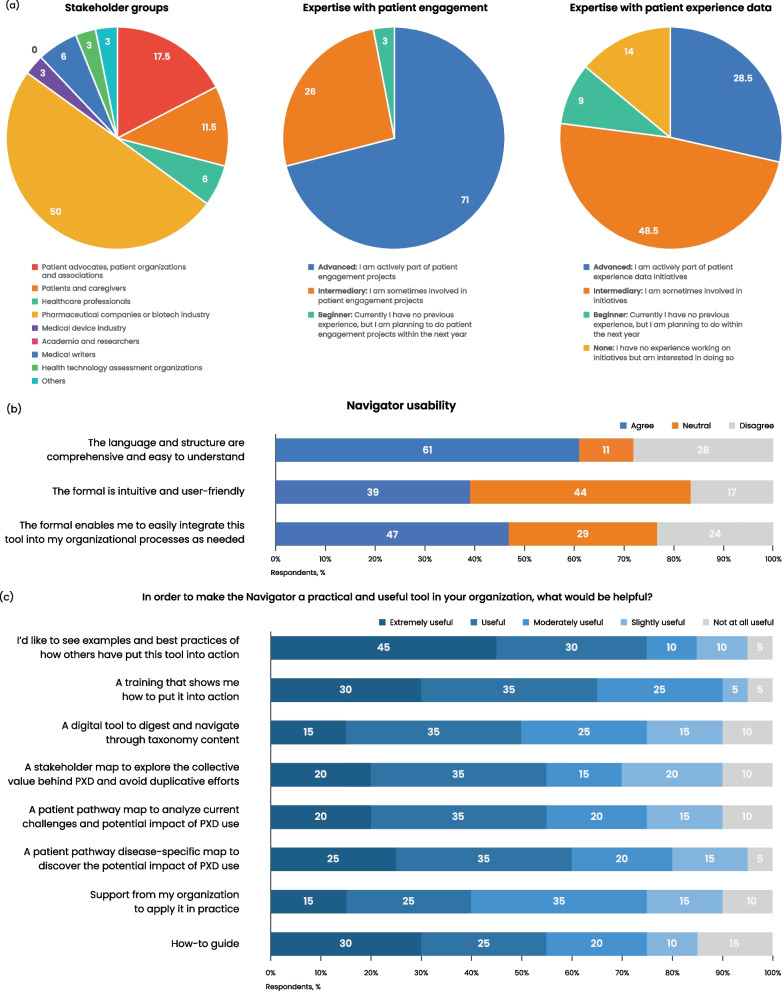

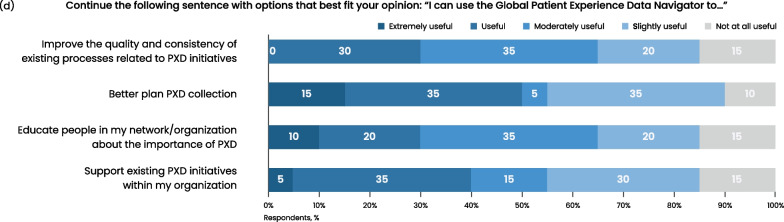
Results of the public consultation survey. **a** Respondent demographics shown as a percentage of respondents in terms of stakeholder groups represented, expertise with patient engagement, and expertise with patient experience data (N = 35). **b** Bar graph showing the results of the questions asked around usability of the Global Patient Experience Data Navigator. Respondents chose agree, neutral, or disagree in relation to the given statements (N = 18). **c** Bar graph showing respondents’ opinions regarding the relative usefulness of the Navigator for PXD initiatives (N = 20). **d** Bar graph showing respondents’ ranking of the relative value of ways to help make the Navigator a practical tool (N = 20). *PXD* patient experience data

Phase 6.5 was aimed at gathering public insights and feedback on the user experience of the Global Patient Experience Data Navigator from a wide audience. Thirty-five participants from 13 countries across eight stakeholder groups took part in a public online survey-based consultation (Fig. 4). Patient advocates and patient organizations and associations comprised 17.5% of respondents (Fig. 4a). Of the 35 respondents, 71% reported having an advanced level of expertise in PE projects and being actively involved in such projects within their organizations, 26% had intermediary expertise and were sometimes involved in PE projects, and 3% said they were beginners and had no experience but were planning to participate in PE activities in the coming year. However, when respondents were questioned about their level of expertise with PXD, only 28.5% self-reported having an advanced level of expertise and being actively involved in PXD initiatives, indicating that PE and PXD are not routinely integrated. Almost half (48.5%) self-reported as intermediary level and as sometimes involved in PXD projects, 9% were beginner level, and 14% had no experience but were interested in being involved in PXD in the future.

The respondents were further questioned about the usability of the Navigator and asked whether they agreed, were neutral, or disagreed with three statements about the language and format of the Navigator (Fig. 4b). The majority (61%) agreed that the language and structure are comprehensive and easy to understand; 11% were neutral, and 28% disagreed. Overall, 39% agreed that the format is intuitive and user-friendly, 44% were neutral, and 17% disagreed. Almost half (47%) agreed that the format enables them to easily integrate this tool into their organizational processes, 29% were neutral, and 24% disagreed.

Respondents were also asked about the relative usefulness of the Navigator for PXD, and to select options that best fit with their opinion of how to finish this sentence: “I can use the Global Patient Experience Data Navigator to…” (Fig. 4c). The majority (85–95%) reported that they found the Navigator useful to some degree (ranging from extremely to slightly useful) for each of the four options of: support existing PXD initiatives within my organization; educate people in my network/organization about the importance of PXD; better plan PXD collection; and improve the quality and consistency of existing processes related to PXD initiatives (Fig. 4c).

Respondents were asked what would help to make the Global Patient Experience Data Navigator a practical and useful tool in their organization and given eight options to rank in terms of usefulness (Fig. 4d). Most people found all the suggestions useful to some degree. The suggestion of examples to see best practices of the tool being used, training to illustrate how to put it into action, and a digital tool to help navigate through taxonomy content were ranked useful to some degree by 95%, 95%, and 90% of respondents, respectively. Ninety percent agreed that a stakeholder map to explore the collective value behind PXD and avoid duplicative efforts would be useful to some degree, with 55% stating this would be either extremely useful or useful. Similarly, most respondents said that a patient pathway map to analyze current challenges and the potential impact of PXD (90%), and a patient pathway disease-specific map to discover the potential impact of PXD (95%), would be useful to some degree. Support from their organization to apply the Navigator in practice was viewed as useful by 90%; 85% thought a “How-to guide” would be useful to some degree, with 55% ranking this as extremely useful or useful.

A plain English definition and examples of specific measurement tools/methods for the impact area were suggested as a useful addition, along with both a disease-specific and disease-agnostic version. One issue raised was the need to clarify the target audience, as it was felt that stakeholders would need to combine their efforts to capture PXD as comprehensively as set out in the Navigator. One respondent also suggested it would be helpful to extend the role of patients, particularly expert and independent patients who are advocates for their health community, in PXD development and collection to the point of shared leadership with other stakeholders.

## Discussion

The Global Patient Experience Data Navigator was made available on the PFMD website in December 2022 and consists of an introductory guide, four tools that can be downloaded in the form of templates, and a glossary to aid implementation. In addition, the Navigator helps users select the appropriate means by which to measure outcomes that matter most to them, identify which stakeholders are using PXD and when, and consider the impact of these data on healthcare decision-making.

The Navigator was designed through a diverse, multi-stakeholder collaboration to create a practical tool that is globally applicable, valid across different health conditions, and can be tailored for a range of PXD projects. The phased, co-creation approach to develop the Navigator was selected based on previous experience with the development of PE quality guidance [15] as a robust and inclusive methodology for multi-stakeholder collaboration and incorporation of public and patient perspectives. The collective input from the diverse WGs aimed to ensure that the tool is as widely applicable and relevant as possible, and the smaller and individual meetings that took place alongside this provided additional opportunities for refinement. The co-creation method enabled a wider understanding, helped to drive decision-making, and assisted with the implementation of the insights/concepts discussed in the meetings. The final output of tables that form the basis of the current toolset builds upon previously published taxonomies using the expertise of the WG and SC members (almost half of which were patient representatives) to include additional categories [9, 17].

Significantly, the Global Patient Experience Data Navigator builds on previously published taxonomies, but with a paradigm shift that identifies “meaningful PXD” as defined by patients, which has not been addressed to date. In addition, the Navigator is the first tool to provide publicly available templates for PXD generation and collection through a coherent and integrated patient-centered model as opposed to individual product-specific or development-phase–specific approaches.

TransCelerate Biopharma has developed a Patient Protocol Engagement Toolkit to engage patients early in clinical trial protocol design, along with a Study Participant Feedback Questionnaire to assess patient experiences during clinical studies [20, 21]. However, these toolkits differ in scope and structure from the Navigator. They are formulated as questionnaires specifically focused on taking the relevant stakeholders through all the necessary steps to establish a partnership with patients. They focus on evaluating the experience of patients in the context of PE (e.g., quality of the engagement, logistics, impacts) rather than collecting PXD. They are specifically designed for clinical trials to help maximize PE in terms of protocol development, and to gather feedback during the trials. While complementary to any current resources, the Navigator differs as it is a taxonomically grouped resource for the collection and organization of PXD for patients with a disease or a treatment and aims to better understand the impacts these have on their lives.

A Patient Experience Toolkit has been co-designed and evaluated in a study to establish how healthcare personnel can use PXD to improve future care delivery [22]. However, the toolkit was not made publicly available. It was solely used for the purpose of the study, which concluded that the current collection of PXD is often “not fit for purpose” in enabling healthcare providers to make meaningful improvements. The authors noted that the diverse settings in which the study took place was a limitation and that the toolkit required “skilled facilitation” to achieve a successful outcome.

The public consultation collected preliminary data to indicate how the Global Patient Experience Data Navigator is perceived, and will be received, by prospective users, and indicates that, overall, stakeholders see value in what the toolkit offers for various PXD projects. However, we acknowledge that there are limitations to the public consultation phase, including the small sample size and the lack of expertise of many respondents regarding PXD, which possibly limited their ability to make granular choices in terms of ranking usefulness and suggesting ways to make the tool more practical. Nevertheless, insights from this survey have indicated a need for simplified language and structure, and that an introductory guide giving examples of how the tools might be used would be a valuable addition to the toolkit. General comments also suggested that additional guidance, training, and education may be needed to understand how best to implement the Navigator and how it might be used for decision-making. Feedback from the consultation has helped to determine the project's strategic direction in terms of making it more publicly accessible, and further feedback will be considered to improve user experience and adaptability of the Navigator as the project progresses.

To aid the implementation of the Navigator and to help orientate users, a glossary of commonly used terms in PE and PXD was also developed. The glossary is currently published as part of the Navigator but continues to be refined as terms and definitions evolve. Preliminary feedback on the glossary indicates it is a useful addition, but more simplified definitions are required. Therefore, continuing this work will be a focus of the project going forward.

In general, lack of knowledge about the collective value of PE and PXD initiatives is a challenge in this field and was specifically identified in the Patient Engagement Open Forum discussion as a systemic barrier (see Additional file 5: Table 2). This will be addressed in future work on this project, which will focus on educational initiatives to help stakeholders understand how they can apply the tools, for example, by providing training, case studies/working examples, repositories, templates, and links to useful resources.

Several pilot studies using the Global PED Navigator are currently underway. One example provided by IPSEN, is an initiative to gather PED for disease-specific patient journeys by mapping both patient and caregiver experiences in different disease contexts, including medical (diagnosis-/treatment-related experience), emotional/psychological, social experience and daily functioning, and health-related quality of life. It also uses a selection of data capture methods and outcomes assessment strategy distributed throughout the patient journey, covering symptoms, diagnosis, and treatment, along with other useful measures. A second study, led by the International Alliance of Dermatology Patient Organizations (GlobalSkin)—the Global Research on the Impact of Dermatological Diseases (GRIDD)—focused on measuring and improving the quality of life for patients with dermatological conditions. Patients volunteered to be part of the development of a measurement tool which to date has collected data regarding each dermatological condition, demographics, along with healthcare-related questions and patient-reported measures (such as the new Patient-Reported Impact of Dermatological Diseases (PRIDD) [impact of dermatological conditions], EQ-5D [generic quality of life], WHO-5 [mental well-being], PHQ9 [depression], GAD7 [anxiety], and DLQI [dermatology quality of life]). This did not initially use the Navigator, but upon retrospective consideration, the researchers have commented it would have provided valuable guidance and structure. The Navigator's approach to capturing PXD aligns with the methods already employed in this initiative and would have helped to reaffirm the validity of the chosen data collection methods.

We acknowledge that in terms of the development process taken, there were some limitations. The discussions to achieve consensus were time-consuming and might have benefited from a more directed approach from the outset, such as a set number of meetings and, for certain elements, voting to achieve consensus. In addition, the project could benefit from increasing the diversity of the participants in terms of those on the WG and SC and is something we are working towards for future projects. Additional limitations, as mentioned, concerned document language and structure, which prospective users found could be more user-friendly and comprehensive.

Aside from the development of the Navigator itself, the PE and PXD project and the general taxonomy research have helped to engage and empower stakeholders. The research has helped to inform current and future initiatives, both directly through the use of the Navigator and indirectly through the many meetings and engagement initiatives, by demonstrating how much can be achieved and by driving further PE and advocacy.

## Conclusions

The Global Patient Experience Data Navigator provides a comprehensive toolset for diverse stakeholders to undertake strategically planned PXD collection, making it coherent, patient-centered, and integrated. Following the public consultation that helped inform the project’s strategic direction, future work will focus on integrating the Navigator into a PE context and ensuring users understand how to engage patients in the generation and use of PXD. This will be supported through guidance, education, and training, to improve and maximize PE and PXD initiatives.

### Supplementary Information

**Additional file 1**. GRIPP2-SF checklist.

**Additional file 2**: Table S1. Working group and steering committee members and their affiliations.

**Additional file 3**: Table S2. Key resources used for the development of the Global Patient Experience Data Navigator.

**Additional file 4: Table 1**. Summary of working group meetings and key outputs.

**Additional file 5: Table 2**. Development process: phase 6—validation and formalization expanded view.

## Data Availability

The datasets used and analysed during the current study are not publicly available due to the small dataset, but specific data is available from the corresponding author upon reasonable request.

## Abbreviations

ClinROs: Clinician-reported outcomes
COA: Clinical outcome assessment
FAQs: Frequently asked questions
FDA: US Food and Drug Administration
HTA: Health technology assessment
NHC: National Health Council
ObsROs: Observer-reported outcomes
PC-CIS: Patient-centered core impact sets
PPI: Patient and public involvement
PE: Patient engagement
PerfROs: Performance outcomes
PFMD: Patient Focused Medicines Development
PROs: Patient-reported outcomes
PXD: Patient experience data
SC: Steering committee
WG: Working group
