# Supplementary material for: Co-creation of the Global Patient Experience Data Navigator: a multi-stakeholder initiative to ensure the patient voice is represented in health decision-making
Source: Res Involv Engagem. 2023 Oct 12;9:92. doi: 10.1186/s40900-023-00503-9 (PMC10571339; doi:10.1186/s40900-023-00503-9)
Supplement: Supplementary file 1 — Additional file 1. GRIPP2-SF checklist. [file 40900_2023_503_MOESM1_ESM.docx]

**GRIPP2-SF checklist**

1. *PPI* patient and public involvement

| **Section and topic** | **Item** | **Reported on page No** |
| --- | --- | --- |
| 1: Aim | Report the aim of PPI in the study | 6 |
| 2: Methods | Provide a clear description of the methods used for PPI in the study | 7, 8 |
| 3: Study results | Outcomes—Report the results of PPI in the study, including both positive and negative outcomes | 8-13 |
| 4: Discussion and conclusions | Outcomes—Comment on the extent to which PPI influenced the study overall. Describe positive and negative effects | 13–16 |
| 5: Reflections/critical perspective | Comment critically on the study, reflecting on the things that went well and those that did not, so others can learn from this experience | 13–16 |
