# Supplementary material for: Co-creation of the Global Patient Experience Data Navigator: a multi-stakeholder initiative to ensure the patient voice is represented in health decision-making
Source: Res Involv Engagem. 2023 Oct 12;9:92. doi: 10.1186/s40900-023-00503-9 (PMC10571339; doi:10.1186/s40900-023-00503-9)
Supplement: Supplementary file 2 — Additional file 2: Table S1. Working group and steering committee members and their affiliations. [file 40900_2023_503_MOESM2_ESM.docx]

**Supplementary Table 1** Working group and steering committee members and their affiliations

| **Name** | **Role and affiliation** | **Stakeholder group represented** |
| --- | --- | --- |
| **Working group members** | | |
| Tom Willgoss | People and Product Leader, Patient Centered Outcomes Research, Roche | Pharma |
| Brett Hauber | Senior Director, Patient Preference Elicitation in Global Medical Impact Assessment, Pfizer; also affiliated with IMI-PREFER | Pharma |
| Carole Scrafton | Director and Co-founder, Flutters and Strutters Patient Advocacy Organisation Patient Advocate | Patient representative |
| Christiana Evers | Vice-President, Chief Community Engagement Officer, Parkinson’s Foundation | Patient representative |
| Conny Berlin | Global Head Quantitative Safety & Epidemiology, Novartis; also affiliated with IMI-PREFER | Pharma |
| Devika Nair | Assistant Professor of Medicine, Vanderbilt University Medical Center | Healthcare professional |
| Eleanor Perfetto | Formerly Executive Vice President of Strategic Initiatives, National Health Council | Patient representative |
| Elisabeth Oehrlein | Formerly Assistant Vice President, Research and Programs National Health Council | Academic/patient representative |
| Erica Spies | Director, Strategy Innovation & Capabilities Global R&D, EMD Serono | Pharma |
| Helene Schoemans | Hematologist, UZ Leuven | Academic |
| Jayne Galinsky | Formerly Head of Patient Evidence, MPE | Academic/patient representative |
| Jessica Scott | President, Legacy Health Strategies | Patient representative |
| Jill Abell | Executive Director, Patient, Caregiver and Consumer Experience, Merck | Pharma/patient experience |
| Julia Tolley | Head of Operations and Business Development, Patvocates | Patient representative |
| Laure Delbeque | Principal Research Scientist, Eli Lilly | Pharma |
| Marilyn Metcalf | Senior Director, Patient Engagement, GSK | Pharma/patient representative |
| Peter Trask | Senior Director Patient Centred Outcomes Research and Oncology TA Head for PCOR, Genentech | Pharma |
| Richie Castles | Formerly Director, Patient Engagement Lead ACE Region, Medical Affairs, Gilead Sciences Europe Ltd | Pharma |
| Sandra Lamy | Associate Director, Oncology, International Regulatory Affairs, Gilead Sciences International | Pharma |
| Sharareh Hosseinzadeh | Formerly Global Head of Scientific PE in Global Drug Development, Novartis | Pharma |
| Silke Schoch | Manager, Research & Programs National Health Council | Academic/patient representative |
| Silvia Ferré | Senior Director, Patient Outcomes Research, National Kidney Foundation | Patient representative |
| Ulrik Kihlbom | Associate Professor in Medical Ethics, IMI-PREFER Representative, Uppsala University | Academic |
| Victoria Livingstone | Associate Director, Global Patient Engagement, Medical Affairs, Gilead Sciences Europe Ltd | Pharma |
| **Steering committee members** | | |
| Alissa Jaffe Nagler | Formerly Executive Director, Patient Engagement Global Functions, Novartis | Pharma |
| Giorgio Barbareschi | Program Manager,  European AIDS Treatment Group (EATG) | Patient representative |
| Jessica Scott | President, Legacy Health Strategies | Patient representative |
| Jim Elliott | Public Involvement Lead, Health Research Authority | Patient representative/health authority |
| Karlin Schroeder | Associate Vice President, Community Engagement, Parkinson’s Foundation | Patient representative |
| Kelli Collins | Director of Patient Engagement, National Kidney Foundation | Patient representative |
| Neil Bertelsen | HTAi Patient And Citizen Involvement Interest Group | Patient representative/health authority |
| Rebecca Vermeulen | Head of Healthcare and Patient Partnership Teams, Roche | Pharma |
| Silvia Ferré | Senior Director, Patient Outcomes Research, National Kidney Foundation | Patient representative |
| Victoria Dohnal | Associate Director Global Regulatory Policy and Intelligence, Janssen | Pharma |
| Victoria Livingstone | Project Manager, Global PE team, Gilead Sciences | Pharma |
