# Supplementary material for: Co-creation of the Global Patient Experience Data Navigator: a multi-stakeholder initiative to ensure the patient voice is represented in health decision-making
Source: Res Involv Engagem. 2023 Oct 12;9:92. doi: 10.1186/s40900-023-00503-9 (PMC10571339; doi:10.1186/s40900-023-00503-9)
Supplement: Supplementary file 3 — Additional file 3: Table S2. Key resources used for the development of the Global Patient Experience Data Navigator. [file 40900_2023_503_MOESM3_ESM.docx]

**Supplementary Table 2** Key resources used for the development of the Global Patient Experience Data Navigator

| **Resource** | **Which section of the Navigator this helped to develop and how** |
| --- | --- |
| (1) FDA Guidance: Patient-Focused Drug Development: Methods to Identify What Is Important to Patients Guidance for Industry, Food and Drug Administration Staff, and Other Stakeholders; February 2022. <https://www.fda.gov/media/131230/download>. Accessed April 14, 2023  (2) FDA government website slide deck: COLLECTING PATIENT EXPERIENCE DATA: HOW YOU CAN BEST HELP FDA? 2022. [https://www.fda.gov/media/112163/download](https://www.fda.gov/media/112163/download.). Accessed April 14, 2023  (3) Chiara Lauren Whichello. PhD Thesis: *Patient Preferences Throughout the Medical Product Lifecycle.* Erasmus University of Rotterdam, 2020. | These sources all supported the development of the first two sections of the Navigator, which examine approaches to identify the outcomes that matter most to patients and families and tools for measuring these outcomes  The evaluation of most appropriate tools using these sources; also supported the heat map development for the first two sections  Definitions contained within source 2, the FDA 2022 report, were used to help develop the glossary |
| (4) Annabel de Maria, Nerea Blanqué (Alira Health website). *Level of Patient Involvement in Health Technology Assessment (HTA) Agencies: A Systematic Literature Review by Alira Health*. December 2021. <https://alirahealth.com/education-hub/patient-involvement-health-technology-assessment-agencies-hta/>. Accessed April 14, 2023  (5) Scott AM, Wale JL. HTAi Patient and Citizen Involvement in HTA Interest Group, Patient Involvement and Education Working Group. Patient advocate perspectives on involvement in HTA: an international snapshot. Res Involv Engagem. 2017;3:2. <https://doi.org/10.1186/s40900-016-0052-9>. Accessed April 14, 2023  (6) Biotechnology Innovation Organization website. <https://archive.bio.org/sites/default/files/docs/toolkit/Product-Lifecycle-Graphic.pdf>. Accessed April 14, 2023  (7) CIOMS Working Group. *Patient involvement in the development,*  *regulation and safe use of medicines* (draft report); February 24, 2022 | These sources all supported development of the third section of the Navigator, which examines the use of PXD within the drug development process, with a specific focus on patient engagement with HTA agencies  Sources 5 and 6 specifically supported the identification of key decision points that might be supported by PE and PXD |
| (8) National Health Council website.  A Blueprint for Developing Patient-Centered Core Impact Sets (PC-CIS). <https://nationalhealthcouncil.org/a-blueprint-for-developing-patient-centered-core-impact-sets-pc-cis/>. Accessed April 14, 2023 | This supported the identification of areas of impact based on the Williamson-Clarke taxonomy. The revised taxonomy was integrated in the first two sections, allowing easier grouping of the areas of impact of a disease or a treatment |
