# Supplementary material for: Co-creation of the Global Patient Experience Data Navigator: a multi-stakeholder initiative to ensure the patient voice is represented in health decision-making
Source: Res Involv Engagem. 2023 Oct 12;9:92. doi: 10.1186/s40900-023-00503-9 (PMC10571339; doi:10.1186/s40900-023-00503-9)
Supplement: Supplementary file 4 — Additional file 4: Table 1. Summary of working group meetings and key outputs. [file 40900_2023_503_MOESM4_ESM.docx]

**Table 1** Summary of working group meetings and key outputs

| **Date/ location** | **Participants, n/N** | **Stakeholders represented** | **Meeting objectives/key outputs** |
| --- | --- | --- | --- |
| September 16, 2021 | 13/21 | 6 patient representatives, 1 regulator, 6 pharma industry representatives | - Established that a global PXD taxonomy would provide a standardized approach for the development and use of PXD and identified four main areas for PXD use and development within this model:   (1) highlight the needs that are most important and meaningful to patients; (2) review tools and methodologies available (and identify gaps) to measure these patient experiences; (3) identify which stakeholders are using PXD and how/when it is being used; (4) consider the impact of these data on healthcare decision-making |
| October 28, 2021 | 9/27 | 4 patient representatives, 5 pharma industry representatives | - Reviewed the content for part 1 of the taxonomy model―addressing what is most meaningful to patients - Consensus was to develop a model that incorporates the areas of impact highlighted in the NHC blueprint for developing PC-CIS [9], with current available tools needed to monitor those impacts - Reviewed the content for part 2 of the taxonomy model―addressing the methodologies and tools available―and agreed to provide stakeholders with clear integrated guidelines based on their evolving needs - The FDA PXD submission table as described by Kieffer et al. [16], which lists tools that could be used to collect PXD, was identified as a useful starting point, noting that the following methods to prioritize what is most important to patients could be added: individual patient/family interviews, focus group interviews, clinician/other stakeholder interviews, Delphi panels/consensus building methods, surveys with both open-ended and closed questions, social media data, patient-preference studies, natural history studies, epidemiological studies, online bulletin boards, patient registries, observational methods, regulatory/HTA-led and externally led engagement meetings - Additional methods to measure what it is most important to patients include the following: qualitative methods, PROs, ClinROs, ObsROs, PerfROs, digital health technologies, biomarkers, real-world endpoints, questionnaires, patient-preferences studies - Agreed that a registry could be easily integrated to collect PXD and other macro-categories, such as PROs and COAs, and that a disease-agnostic approach would help streamline the development process |
| December 10, 2021 | 12/32 | 4 patient representatives, 3 IMI-PREFER representatives, 4 pharma industry representatives | - Continued to build on part 1 of the taxonomy model―addressing what is most meaningful to patients―using the Williamson-Clarke taxonomy [17] and incorporated the six main areas of impact on patients as identified by the NHC: mortality/survival, signs and symptoms (including objective and subjective symptoms), functioning, treatment experience, resource use, patient journey - Part 2 of the taxonomy model―addressing approaches and tools used to measure impact on patients―was drafted and presented by Tom Willgoss^a^ - Based on his experience and FDA guidance, four different approaches, or steps, that could be applied while considering PXD were included: (1) approaches to identify unmet needs and outcomes that matter most to patients and families; (2) tools for measuring the outcomes that matter to patients and families; (3) methods for capturing patients' preferences; (4) methods for generating evidence - Discussed part 3 of the model―addressing the value of PXD for diverse stakeholders in terms of how and when they are using these tools - Concluded that PXD approaches/tools used, and for what objectives, need to be validated by each stakeholder group |
| February 3, 2022 | 14/29 | 5 patient representatives, 2 IMI-PREFER representatives, 7 pharma industry representatives | - Considered progress of the PE and PXD project and how the process has influenced the broader healthcare system - Reviewed the taxonomy model and identified novel PXD tools/methods that might be used - Discussed being aware of the definition of “meaningful impact” as outlined by patients - Selected fit-for-purpose tools to capture a PXD co-created validation strategy - Agreed that further support for content and structure validation was required for part 2 of the taxonomy model developed by Tom Willgoss^a^ - Concluded that parts 3 and 4―focusing on the possible/ideal uses of PXD among stakeholders and healthcare processes―will help define which stakeholders are generating and using PXD. They will also help to identify possible overlap and duplicative efforts, as well as actual PXD use by diverse stakeholders to be integrated with the PXD use pathway to illustrate future opportunities - Agreed that further support was needed to help develop the section addressing “when” PXD would be used and two options were identified: (1) the drug development cycle, which helps to explain which phase of the process PXD could be used in and why; (2) patient touchpoints, which would highlight key steps of patient interactions with the healthcare system during treatment - Overall structure of the taxonomy model was validated |
| March 3, 2022 | 11/31 | 5 patient representatives, 2 IMI-PREFER representatives, 2 pharma industry representatives, 1 regulator, 1 consultant | - Discussed the potential uses of the Global Patient Experience Data Navigator based on the type of stakeholder - Discussed how the Navigator can be used to select relevant information, support informed conversations, empower patients to have more control over the decision-making and drug development process, advocate for these uses and approaches in the development of programs, and gain a deeper understanding of the evidence that needs to be generated to help patients' decision-making - Discussed the need for an introductory document for the Navigator detailing the needs and purpose of the taxonomy, potential use based on audience, structure description, and methodology of development |
| April 20, 2022 | 15/30 | 4 patient representatives, 1 IMI-PREFER representative, 7 pharma industry representatives, 1 regulator, 1 consultant, 1 academic | - Discussed plans for the public consultation, in terms of where and how it should be communicated, what should be assessed and what resources would help to disseminate it - Development of a simplified version was suggested as the content is still deemed too technical for some; a communication toolkit was considered essential, along with different messages for industry and professionals; and it was agreed there should be a clear outline of what is meant by the co-creation process - Potential topics for the public consultation included usability, content, and the potential future development of the taxonomy, with a focus on value for diverse stakeholders. Capturing further information, such as on tools and resources that could enable PXD collection and potential use of taxonomy output, was also suggested |
| June 2, 2022 | 6/30 | 4 patient representatives, 1 pharma industry representative, 1 consultant | - Reviewed the Navigator’s new branding and design structure - Discussed the four potential outcomes for the PE and PXD project in terms of uses and improvements:   (1) The PXD Navigation Tool―inclusion of a case study repository to disseminate best practices; integration of multi-stakeholder perspectives on PXD collection and use; and inclusion of links to PE resources to support the generation and use of PXD  (2) The PXD use and impact map―advocating for multi-stakeholder collaborations through the addition of a heat map that can illustrate the overlapping needs across stakeholders and performing a multi-stakeholder analysis of PXD and its impact  (3) A patient pathway―the creation of alliances to collaboratively work on challenges, and the creation of heat maps on PXD areas  (4) The disease-specific PXD repository―connection to journals, taking advantage of artificial intelligence linguistics/free text (i.e., from PubMed) to keep the repository up to date, and progress from general disease to a set of diseases and, potentially, a specific disease   - Discussed the importance of educating people about the relevance and impact of PXD, creating an incentive system for publishers when it comes to PXD sharing, ensuring that the language used is easy to understand and paying attention to multimedia reporting of user experiences |

^a^Tom Willgoss, People and Product Leader, Patient-Centered Outcomes Research, Roche.
*COA* clinical outcome assessment; *ClinROs* clinician-reported outcomes; *FDA* US Food and Drug Administration; *HTA* health technology assessment; *ObsROs* observer-reported outcomes; *NHC* National Health Council; *PC-CIS* patient-centered core impact sets; *PE* patient engagement; *PXD* patient experience data; *PerfROs* performance outcomes; *PROs* patient-reported outcomes
