# Supplementary material for: Co-creation of the Global Patient Experience Data Navigator: a multi-stakeholder initiative to ensure the patient voice is represented in health decision-making
Source: Res Involv Engagem. 2023 Oct 12;9:92. doi: 10.1186/s40900-023-00503-9 (PMC10571339; doi:10.1186/s40900-023-00503-9)
Supplement: Supplementary file 5 — Additional file 5: Table 2. Development process: phase 6—validation and formalization expanded view. [file 40900_2023_503_MOESM5_ESM.docx]

**Table 2** Development process: phase 6―validation and formalization expanded view

| **Phase** | **Validators and contributors** | **No. of insights/**  **additional data** | **No. of contributors** | **Key objectives/output** |
| --- | --- | --- | --- | --- |
| 6.1 | Working group validation  (Dec 10, 2021, to March10, 2022) | 94 | 8 | - Objective: Discuss Global Patient Experience Data Navigator structure and content - Collected general feedback and validated controversial points on the structure - The main outcome can be recognized as the tables in the current version of the Navigator, with a specific focus on the first two tables |
| 6.2 | PFMD strategic advisory board feedback  (April 5, 2022) | 19 | 39 | - This session answered the following questions:   **How to position the Navigator?**  Share key messages to a “community of champions”, i.e., people willing to take responsibility to communicate information to their peers; leverage conferences, events, or workshops to provide more details; consider publications; provide further explanation/communication about why PE and PXD should be integrated  **What outcomes should be included in a “PE and PXD Roadmap”?**  Pilot specific disease areas to create a template; case studies and examples that highlight PE and PXD best practices; tools for implementing PE and PXD together; repository of existing data and activities of a condition  *Additional information to include* FAQs, more examples of the taxonomy, standardized use of the taxonomy, links to common/shared definitions |
| 6.3 | PEOF session on PE and PXD integration  (April 12, 2022) | 173 | 147 | - This session addressed the following areas:   **Multistakeholder value in PE and PXD integration:**  Better treatment plans for patients based on patient experience; holistic patient-centric documentation of benefit–risk; increased/improved evidence generation to support decision-making for, and with, the patient   - **Barriers to PE integration into PXD evidence design and generation:**   Lack of knowledge on methodology; not starting patient engagement in a timely and systematic manner; not aware of what is expected from regulatory and HTA bodies   - **Support, tools and resources needed to strengthen the use/implementation of PE and PXD fusion:**   Education and training; sharing of concrete examples where this has been used and accepted by regulatory and other stakeholders**;** foster the “early and often” inclusion of patient input throughout the development cycle |
| 6.4 | PFMD strategic advisory board feedback  (June 1, 2022) | 80 | 28 | - The PE and PXD fusion workshop was structured using World Café methodology, allowing all groups to provide insights; it focused on identifying opportunities and improvements for the overall strategic direction of the project - The strategic advisory board discussed key points regarding the four potential outcomes of the PE and PXD roadmap, as follows: - A digital tool to identify areas of impacts, relevant tools, and potential impacts: could be further integrated with case studies, standard questionnaires or metrics, and resources - A PXD impact and use map to illustrate the collective value of PXD collection: could be enriched by an overview of the engagement level of the different stakeholders, to improve PXD collection and use in the current context - A disease-agnostic patient pathway to illustrate systemic challenges and PXD potential impact: could support the creation of alliances to collaboratively work on challenges - A disease-specific PXD repository could be linked to the third outcome and provide a progression from a “general” (disease-agnostic) to “specific” (disease-specific) perspective |
| 6.5 | Public consultation  (June 10 to September 17, 2022) | 124 | 36 | - The public consultation aimed to obtain feedback on the Navigator from a wider group on user interaction in terms of language, structure, and how easy it is to integrate into existing processes, as well as potential uses and additional supportive tools (see Fig. 3) |
| 6.6 | PEOF session on PE and PXD integration  (October 4, 2022) | 22 (summarized) | 32 | - The goal of the insights-gathering activity was to review the summary of the How-to guide [18] on Patient Engagement in the Development of a COA Strategy and reflect on recommendations to engage patients in the generation and use of all types of PXD, beyond COAs - A group was assigned to each of four stages: (1) designing and planning; (2) implementation and generation of PXD; (3) analysis and interpretation; (4) communication and use of PXD. All were facilitated by one of the panellists and each group shared ideas about PE at the assigned stage, including for co-creation/creation and for contextualization of data - The face-to-face PEOF session informed the operational direction of phase 2 of the study, suggesting concrete strategies to further integrate PE in PXD planning, generation, analysis, and communication processes |

*COA* clinical outcome assessment; *FAQs* frequently asked questions; *HTA* health technology assessment; *PE* patient engagement; *PXD* patient experience data; *PEOF* Patient Engagement Open Forum; *PFMD* Patient Focused Medicines Development
